# Genome-Wide Characterization of the Myostatin Gene (MSTN) Ligand–Receptor System and Its Expression Patterns Associated with Growth Variation in *Trachinotus ovatus*

**DOI:** 10.3390/ijms27188061

**Published:** 2026-09-10

**Authors:** Jia-Mei Zhou, Hua-Yang Guo, Teng-Fei Zhu, Lin Xian, Bao-Suo Liu, Nan Zhang, Ke-Cheng Zhu, Dian-Chang Zhang

**Affiliations:** 1 Key Laboratory of South China Sea Fishery Resources Exploitation and Utilization, Ministry of Agriculture and Rural Affairs, South China Sea Fisheries Research Institute, Chinese Academy of Fishery Sciences, 231 Xingang Road West, Haizhu District, Guangzhou 510300, China; 2 Sanya Tropical Fisheries Research Institute, Sanya 572018, China; 3 Shenzhen Base of South China Sea Fisheries Research Institute, Chinese Academy of Fishery Sciences, Shenzhen 518121, China; 4 Guangdong Provincial Engineer Technology Research Center of Marine Biological Seed Industry, Guangzhou 510300, China; 5 Guangxi Key Laboratory of Marine Environmental Science, Guangxi Academy of Marine Sciences, Guangxi Academy of Sciences, Nanning 530007, China

**Keywords:** myostatin, activin receptor, *Trachinotus ovatus*, growth regulation, tissue expression

## Abstract

Myostatin (MSTN), a member of the transforming growth factor-β (TGF-β) superfamily, is a conserved negative regulator of muscle growth in vertebrates. In this study, nine MSTN-related genes were identified in *Trachinotus ovatus*, including two MSTN ligand genes (*ToMSTNa* and *ToMSTNb*) and seven putative receptor genes (*ToACVR1A*, *ToACVR1Ba*, *ToACVR1Bb*, *ToACVR2Aa*, *ToACVR2Ab*, *ToACVR2Ba*, and *ToACVR2Bb*). Comprehensive evolutionary and expression analyses revealed the conservation and potential functional diversification of MSTN-related genes in *T. ovatus*. The two MSTN ligands showed relatively conserved gene structures and motif compositions, whereas the receptor genes displayed greater structural diversity. Tissue expression analysis showed that *ToMSTNa* was relatively highly expressed in muscle, while *ToMSTNb* was relatively highly expressed in brain, suggesting potential tissue-specific functional divergence between the two MSTN paralogs. In addition, expression analysis between large and small individuals showed that *ToMSTNa* was more highly expressed in the brain and intestine of small individuals than in large individuals, whereas *ToACVR1A* and *ToACVR2Bb* showed higher expression levels in the liver of small individuals, suggesting their potential association with growth variation. The homology-based predicted protein association network suggested potential associations of ACVR1B- and ACVR2A-type receptors with components of MSTN-related signaling. Overall, this study provides new insights into the evolutionary conservation, tissue-specific functional divergence, and potential growth-associated roles of the MSTN ligand–receptor system in *T. ovatus*, providing a foundation for further functional studies of MSTN-related signaling and the identification of candidate molecular targets associated with growth traits in teleost fish.

## 1. Introduction

MSTN, also known as growth differentiation factor 8 (GDF8), is a member of the TGF-β superfamily and functions as a conserved negative regulator of skeletal muscle growth in vertebrates [1]. Since its first identification in mammals, MSTN has attracted considerable attention because loss of MSTN function can lead to increased muscle mass through both muscle fiber hyperplasia and hypertrophy. In aquaculture species, skeletal muscle growth is directly associated with edible yield and economic value [2]. Therefore, MSTN has been regarded as an important candidate gene for growth regulation and genetic improvement.

MSTN is synthesized as an inactive precursor protein composed of a signal peptide, an N-terminal propeptide region, a proteolytic processing site, and a C-terminal mature domain enriched with conserved cysteine residues, which are essential for dimer formation and biological activity [3]. The N-terminal propeptide maintains MSTN in a latent state before activation, whereas the C-terminal mature peptide is responsible for receptor binding and downstream signaling [3]. After activation, mature MSTN preferentially binds activin type II receptors, mainly *ACVR2A* or *ACVR2B*, and subsequently recruits type I receptors such as ACVR1B/ALK4 to form an active receptor complex [4]. These receptors are transmembrane serine/threonine kinases containing extracellular ligand-binding regions and intracellular kinase domains, thereby enabling ligand recognition and signal transduction [5]. Activation of the MSTN receptor complex induces Smad2/3 signaling and suppresses myoblast proliferation and differentiation, thereby contributing to the regulation of skeletal muscle development and growth [6]. Therefore, both MSTN ligands and their receptor components are essential for understanding MSTN-related signaling pathways involved in vertebrate growth regulation.

Compared with mammals, teleost fish usually exhibit more complex MSTN gene organization and expression patterns. Two or more mstn copies, commonly referred to as *mstna*/*mstn1* and *mstnb*/*mstn2*, have been identified in many fish species, such as gilthead seabream, Atlantic salmon, channel catfish, and rainbow trout [7,8,9]. These paralogs often display distinct tissue distributions and regulatory patterns. For example, Nile tilapia mstn2 is mainly expressed in the brain rather than in red or white muscle, supporting the existence of non-muscular MSTN functions in fish [10]. Functional studies have further demonstrated that MSTN disruption or inhibition can promote muscle growth in several aquaculture species. MSTN-deficient medaka exhibited a double-muscling phenotype characterized by both muscle fiber hyperplasia and hypertrophy, while CRISPR/Cas9-mediated MSTN editing increased muscle cell number and body weight in channel catfish [11,12]. Similar growth-promoting effects have also been reported in genome-edited yellow catfish carrying MSTN mutations [13]. These findings indicate that MSTN-related genes are closely associated with growth regulation in fish and may undergo functional divergence following gene duplication.

*T. ovatus* is an economically important marine fish widely cultured in coastal southern China because of its rapid growth, desirable flesh quality, and high nutritional value [14,15]. As growth performance is one of the most important economic traits in golden pompano aquaculture, elucidating the molecular basis underlying growth regulation is of considerable significance for breeding and production. Although MSTN has been extensively studied as a growth-related factor in vertebrates, systematic information on MSTN ligands and their putative receptor genes is limited in *T. ovatus*, particularly with respect to their structural characteristics, evolutionary relationships, tissue distribution patterns, and potential association with growth variation.

The main objective of this study was to identify and characterize the MSTN ligand–receptor system in *T. ovatus* and to investigate its evolutionary features, tissue-specific expression patterns, and potential association with growth variation. By integrating information on MSTN ligands and their putative receptors, this study aimed to improve our understanding of the organization and potential functional diversification of MSTN-related signaling in teleost fish.

## 2. Results

### 2.1. Genome-Wide Identification and Characterization of MSTN-Related Genes in T. ovatus

A total of nine MSTN-related genes were identified in the *T. ovatus* genome, including two MSTN ligand genes (*ToMSTNa* and *ToMSTNb*) and seven putative receptor genes (*ToACVR1A*, *ToACVR1Ba*, *ToACVR1Bb*, *ToACVR2Aa*, *ToACVR2Ab*, *ToACVR2Ba*, and *ToACVR2Bb*). The predicted physicochemical properties of their encoded proteins are summarized in Table 1. The lengths of these proteins ranged from 364 to 529 amino acids, and their molecular weights varied from 40,895.25 Da (*ToMSTNa*) to 59,593.10 Da (*ToACVR2Aa*). The theoretical isoelectric points (pI) ranged from 5.72 (*ToACVR2Ba*) to 9.06 (*ToACVR2Bb*), with three proteins showing pI values above 7. The grand average of hydropathicity values ranged from −0.602 to −0.161, indicating that all MSTN-related proteins were hydrophilic. In addition, the aliphatic index ranged from 72.22 (*ToACVR2Bb*) to 91.47 (*ToACVR1Bb*). Subcellular localization analysis predicted that *ToMSTNa* and *ToMSTNb* were mainly localized in the nucleus, whereas the putative receptor proteins showed diverse predicted localization patterns, including the mitochondria, plasma membrane, cytoplasm, and extracellular region. As these results were derived from in silico prediction, their actual subcellular localization requires further experimental validation.

### 2.2. Gene Structure Analysis of MSTN-Related Genes in T. ovatus

**Figure 1:**
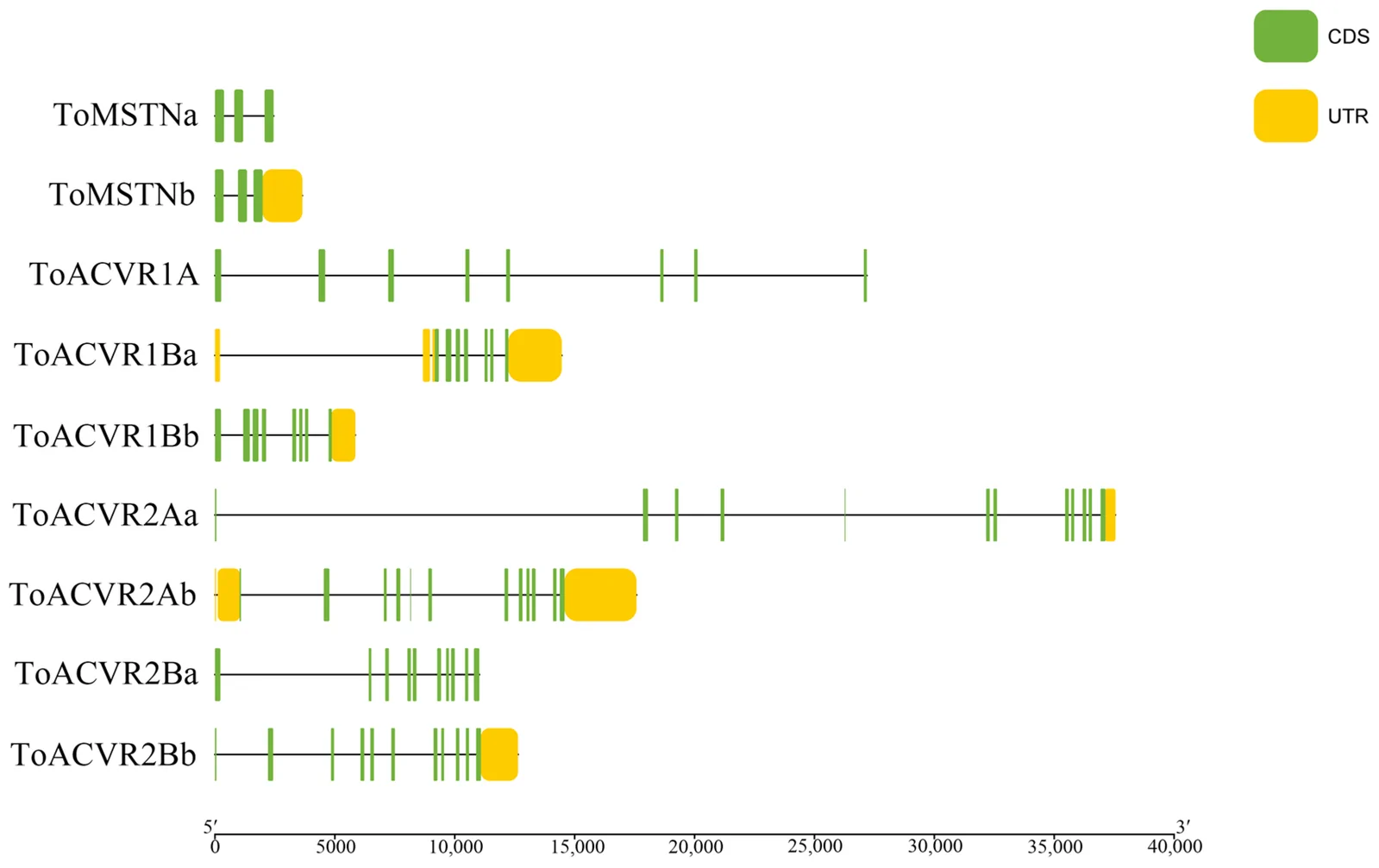
Gene structure analysis of MSTN-related genes in *T. ovatus*. Coding sequences (CDSs) are shown in green, and untranslated regions (UTRs) are shown in yellow.

Among the nine MSTN-related genes identified in *T. ovatus*, *ToACVR1Ba* and *ToACVR2Ab* contained both the 5′ UTR and 3′ UTR, together with complete coding sequences, indicating that these two genes were annotated as full-length gene models (Figure 1). In contrast, *ToMSTNb*, *ToACVR1Bb*, *ToACVR2Aa*, and *ToACVR2Bb* contained only one predicted UTR. To further characterize the structural features of these MSTN-related genes, their exon–intron organization was analyzed. The two MSTN genes, *ToMSTNa* and *ToMSTNb*, displayed relatively compact structures, each containing three coding regions. By contrast, the putative receptor genes showed more complex and variable exon–intron arrangements. Among them, *ToACVR2Aa* spanned the longest genomic region, extending to nearly 40 kb, while *ToACVR1A* also contained a long intronic region. Overall, the MSTN ligand genes exhibited relatively conserved and compact structures, whereas the receptor genes showed greater structural diversity in *T. ovatus*.

### 2.3. Conserved Motifs and Domains of MSTN-Related Proteins in T. ovatus

**Figure 2:**
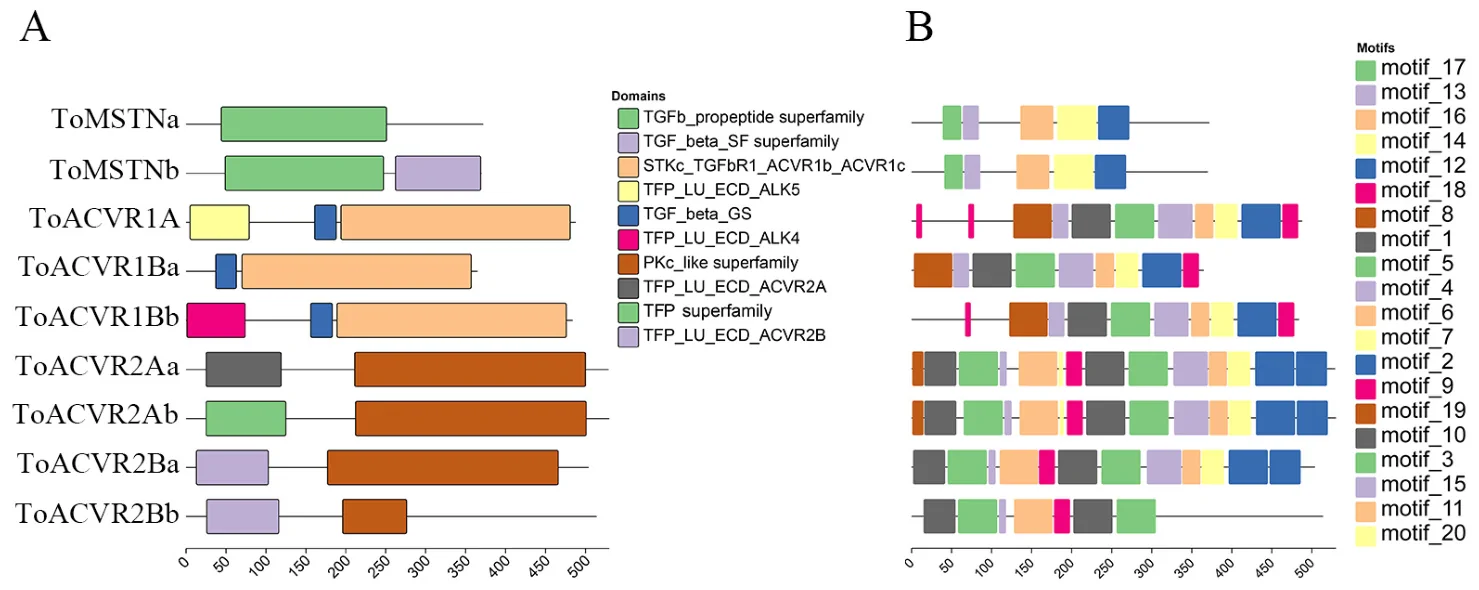
Conserved motif and domain patterns of MSTN-related proteins in *T. ovatus*. (**A**) Schematic representation of conserved motifs in MSTN-related proteins. A total of 20 conserved motifs were identified using MEME. (**B**) Conserved domain organization of MSTN-related proteins in *T. ovatus*.

A total of 20 conserved motifs were identified in the MSTN-related proteins of *T. ovatus* (Figure 2A). Analysis indicated that members within the same subgroup generally exhibited similar motif compositions, suggesting conserved structural characteristics. For instance, *ToMSTNa* and *ToMSTNb* shared highly similar motif patterns, containing motifs 17, 13, 16, 14, and 12. Except for *ToACVR2Ab*, receptor proteins shared several common motifs, such as motifs 1, 3, 4, 6, 7, 9, and 10, indicating relatively conserved motif features among the putative receptor members. Further analysis of conserved domains revealed clear differences between MSTN ligands and their putative receptors (Figure 2B). *ToMSTNa* and *ToMSTNb* both contained the TGFb_propeptide superfamily domain. The receptor proteins contained typical receptor-related domains. Members of the ACVR1 subgroup shared TGF_beta_GS and STKc_TGFbR1 domains, whereas members of the ACVR2 subgroup contained the PKc_like superfamily domain.

### 2.4. Chromosomal Distribution and Synteny Analysis

**Figure 3:**
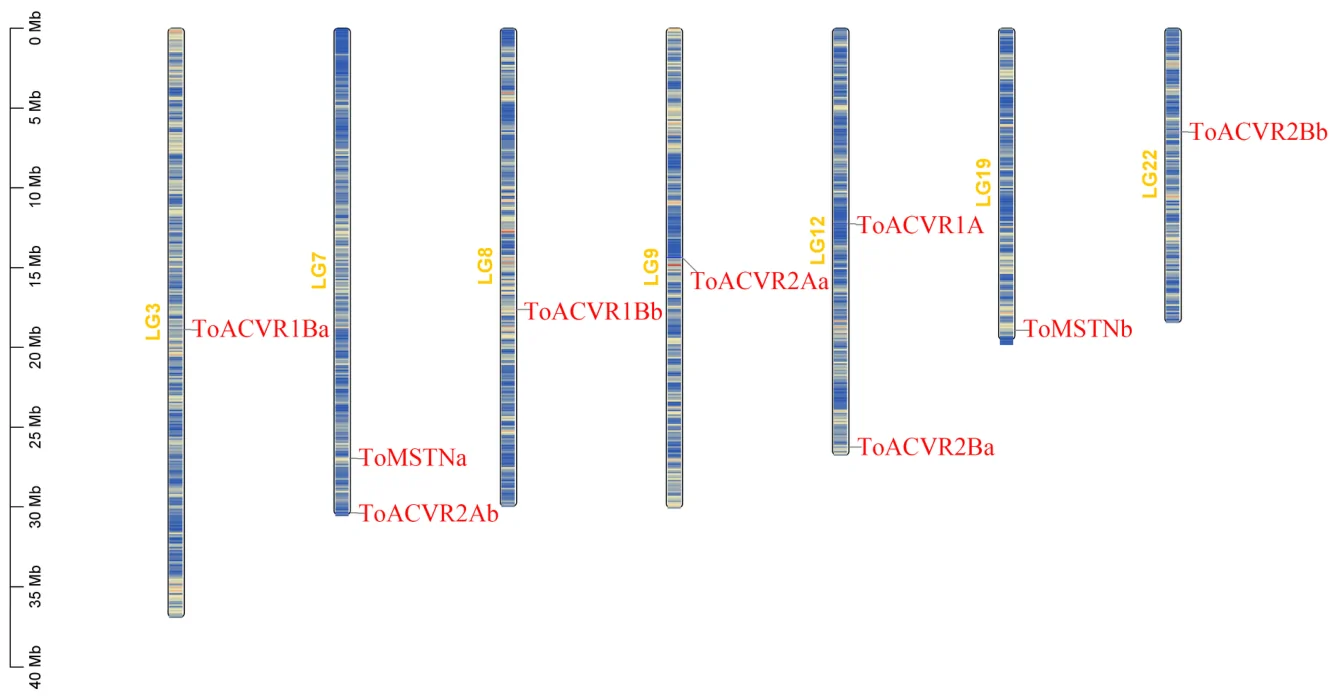
Chromosomal locations of MSTN-related genes in *T. ovatus*. The chromosome length is indicated by the scale bar on the left. Gene names point to the approximate physical positions of MSTN-related genes on each chromosome. “LG” refers to ”linkage group”, corresponding to chromosome numbering in *T. ovatus*. MSTN-related gene names are shown in red, and linkage group (LG) numbers are shown in yellow.

**Figure 4:**
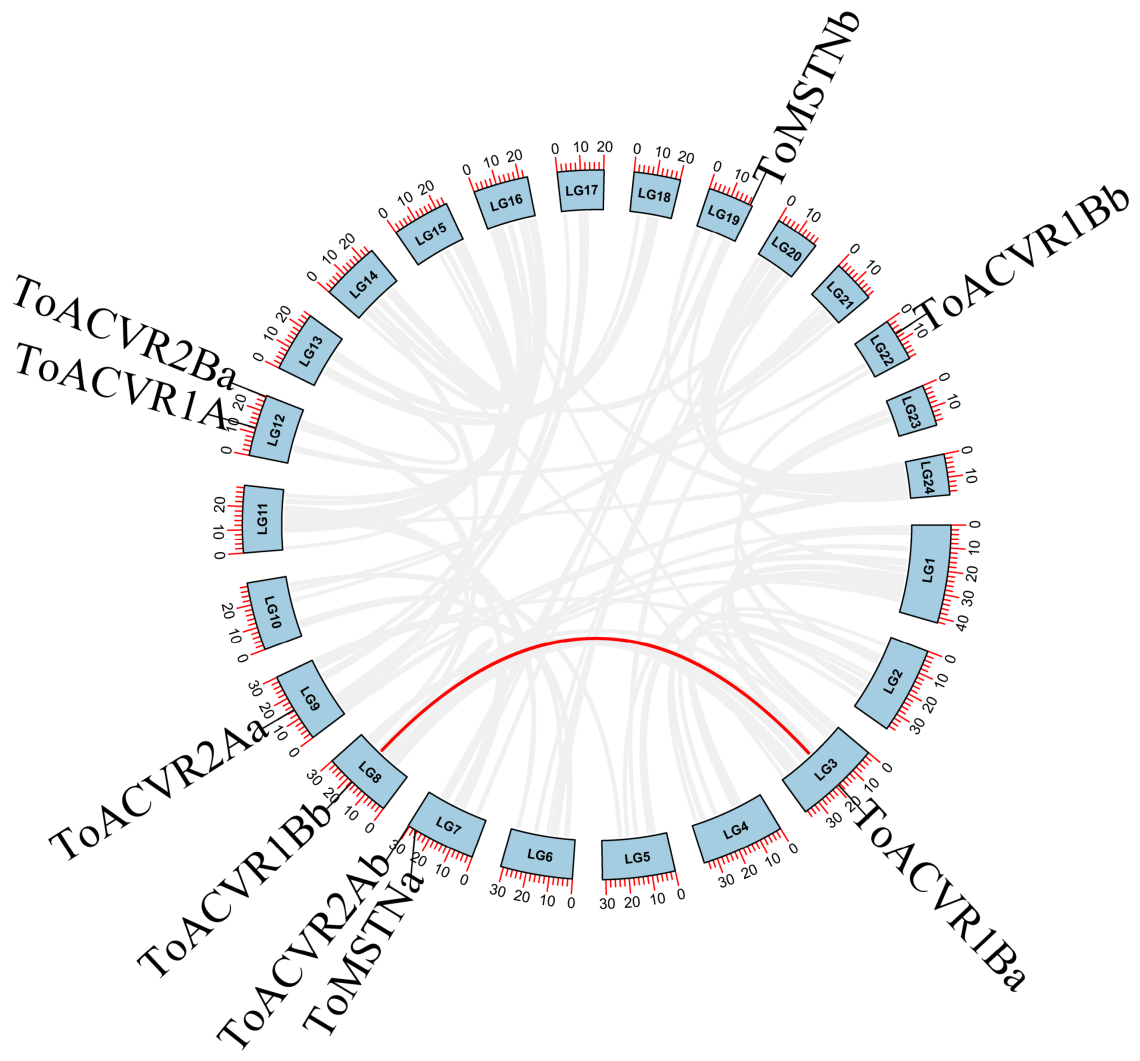
Intraspecific synteny analysis of MSTN-related genes in *T. ovatus*. Gray lines represent all syntenic blocks in the *T. ovatus* genome, and red lines indicate duplicated MSTN-related gene pairs.

**Figure 5:**
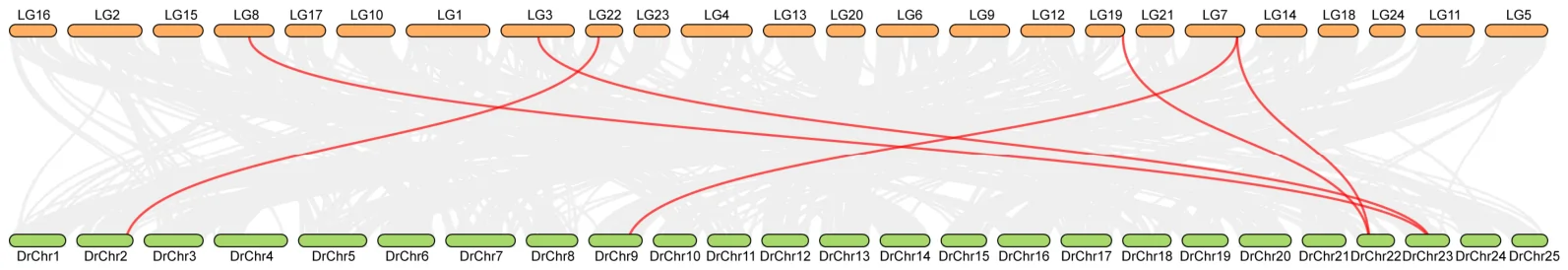
Interspecific synteny analysis of MSTN-related genes between *T. ovatus* and zebrafish. Gray lines represent all syntenic blocks between the *T. ovatus* and zebrafish genomes, and red lines indicate conserved syntenic relationships of MSTN-related genes between the two species.

The nine MSTN-related genes were unevenly distributed across seven chromosomes of *T. ovatus* (Figure 3). Among these chromosomes, LG7 and LG12 each contained two MSTN-related genes, whereas each of the remaining chromosomes harbored only one gene. Intraspecific synteny analysis identified one collinear gene pair, *ToACVR1Ba*–*ToACVR1Bb*, among the MSTN-related genes in *T. ovatus* (Figure 4). This collinear relationship was detected between genes located on LG3 and LG8, suggesting that segmental duplication may have contributed to the retention and diversification of ACVR1B paralogs in *T. ovatus*. Interspecific synteny analysis between *T. ovatus* and zebrafish showed that six MSTN-related genes in *T. ovatus* had conserved syntenic relationships with zebrafish homologs (Figure 5). These genes were distributed on LG3, LG7, LG8, LG9, LG12, LG19, and LG22 in *T. ovatus* and corresponded to homologous regions on zebrafish chromosomes.

### 2.5. Phylogenetic Analysis of MSTN-Related Genes

**Figure 6:**
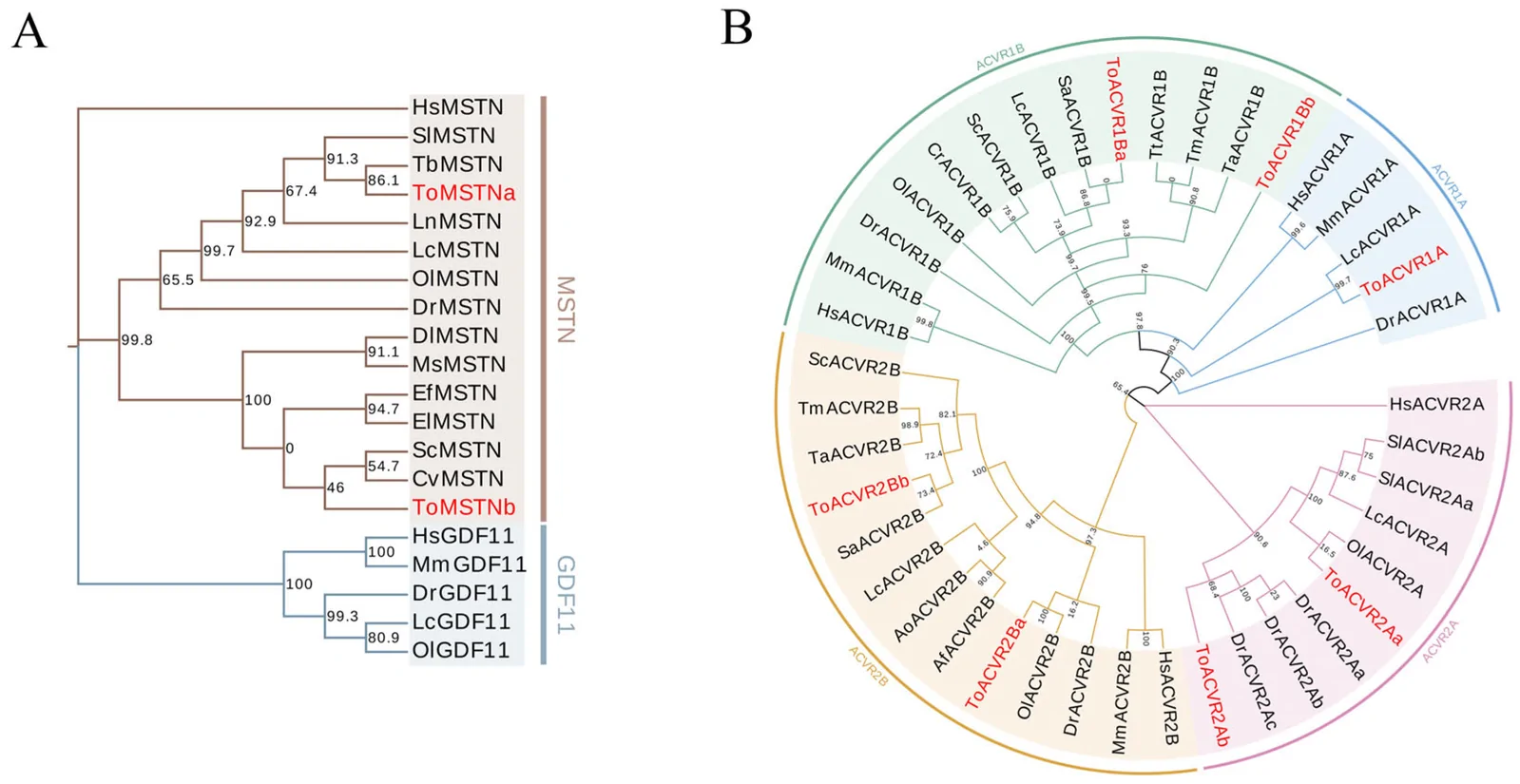
Phylogenetic analysis of MSTN ligands and their putative receptors from different species. (**A**) Phylogenetic tree of MSTN ligand proteins. (**B**) Phylogenetic tree of putative receptor proteins. Different clades are indicated by different colors. MSTN-related proteins from *T. ovatus* are highlighted in red. The species names and sequence information used for phylogenetic tree construction are listed in Table S1.

To further verify the annotation and evolutionary relationships of MSTN ligands and their putative receptors in *T. ovatus*, two phylogenetic trees were constructed. Multiple sequence alignments of MSTN ligands and their putative receptors are shown in Figures S1 and S2, respectively. In the MSTN/GDF11 phylogenetic tree, all sequences were clearly divided into two major clades, corresponding to the MSTN and GDF11 groups (Figure 6A). The two *T. ovatus* MSTN proteins clustered within the MSTN clade, supporting their annotation as MSTN ligands. In the receptor phylogenetic tree, the identified receptor proteins were classified into four subgroups: ACVR1A (*ToACVR1A*), ACVR1B (*ToACVR1Ba* and *ToACVR1Bb*), ACVR2A (*ToACVR2Aa* and *ToACVR2Ab*), and ACVR2B (*ToACVR2Ba* and *ToACVR2Bb*) (Figure 6B). These results support the reliability of MSTN-related gene identification and nomenclature in *T. ovatus*.

### 2.6. Tissue Distribution and Growth-Associated Expression Patterns of MSTN-Related Genes

#### 2.6.1. Basal Expression Patterns of MSTN-Related Genes in Adult Tissues

**Figure 7:**
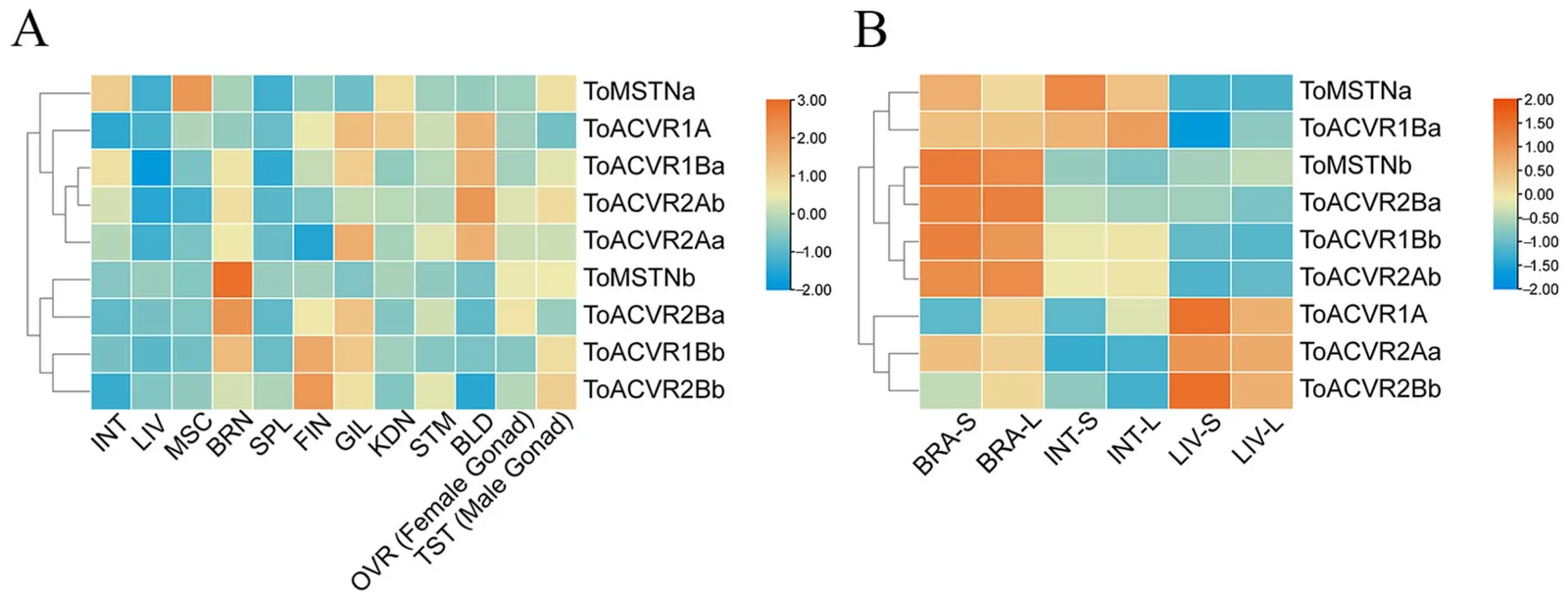
(**A**) Heatmap showing the expression patterns of MSTN-related genes in various healthy tissues of *T. ovatus*. Three biological replicates were included for each tissue (*n* = 3). INT, intestine; LIV, liver; MSC, muscle; BRN, brain; SPL, spleen; FIN, fin; GIL, gill; KDN, kidney; STM, stomach; BLD, blood; TST, testis; OVA, ovary. (**B**) Heatmap showing the expression patterns of MSTN-related genes in the brain, intestine, and liver of large and small individuals of *T. ovatus*. Six biological replicates were included for each growth-performance group (*n* = 6; L1–L6: large individuals; S1–S6: small individuals). BRA, brain; INT, intestine; LIV, liver. The color scale represents log_2_(FPKM + 1) values followed by gene-wise Z-score normalization. Orange indicates relatively high expression, and blue indicates relatively low expression.

The expression levels of MSTN-related genes in 12 adult tissues, including intestine, liver, muscle, brain, spleen, fin, gill, kidney, stomach, blood, testis, and ovary, were evaluated using FPKM values from transcriptomic data and visualized as a heatmap (Figure 7A, Table S2). In adult tissues, the expression patterns of MSTN-related genes were divergent. Among the two MSTN genes, *ToMSTNa* was highly expressed in muscle, whereas *ToMSTNb* showed relatively high expression in brain. In contrast to the MSTN ligands, the receptor genes displayed more diverse tissue-specific expression patterns, with *ToACVR1A*, *ToACVR1Ba*, *ToACVR2Ab*, and *ToACVR2Aa* showing relatively high expression in blood, *ToACVR2Ba* showing high expression in brain and fin, and *ToACVR1Bb* and *ToACVR2Bb* exhibiting relatively high expression levels in fin and ovary.

#### 2.6.2. Expression Patterns of MSTN-Related Genes in Large and Small Individuals

To further investigate the expression characteristics of MSTN-related genes associated with growth variation in *T. ovatus*, we analyzed their expression levels in the brain, intestine, and liver of large (L) and small (S) individuals (Figure 7B, Table S3). The results showed that overall, *ToMSTNa*, *ToMSTNb*, *ToACVR1Ba*, *ToACVR2Ba*, *ToACVR1Bb*, and *ToACVR2Ab* exhibited relatively high expression levels in the brain of both growth groups, whereas *ToACVR1A*, *ToACVR2Aa*, and *ToACVR2Bb* showed relatively high expression levels in the liver. *ToMSTNa* showed a trend toward higher expression in the brain and intestine of S individuals than in L individuals. Similarly, *ToACVR1A* and *ToACVR2Bb* tended to show higher expression in the liver of S individuals.

### 2.7. Predicted Protein–Protein Interaction (PPI) Network of MSTN-Related Genes

**Figure 8:**
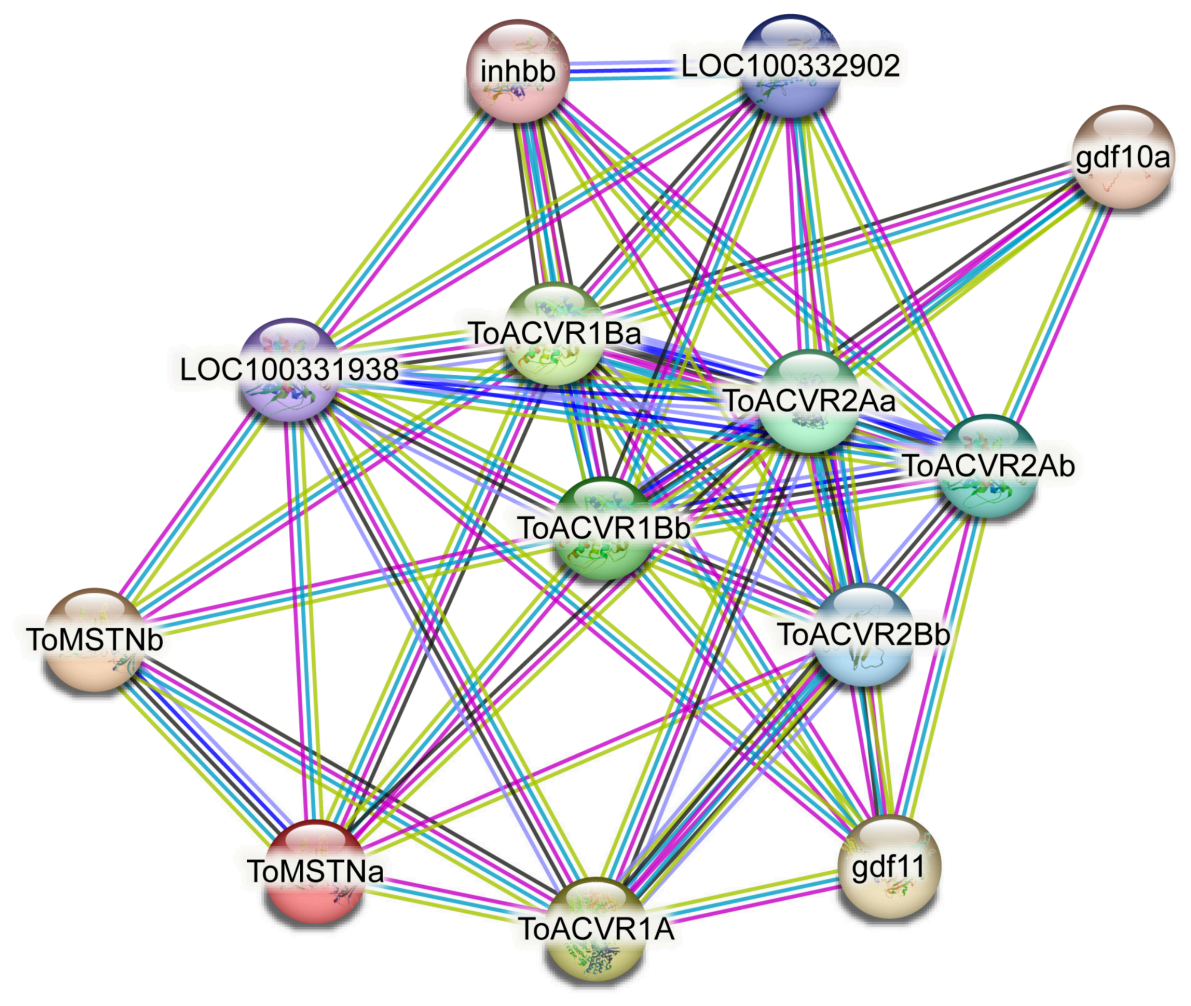
Protein–protein interaction network of MSTN-related proteins in *T. ovatus* predicted using STRING. The network was constructed based on evidence from text mining, experimental data, databases, co-expression patterns, and neighborhood sources, represented by lines of different colors. Nodes with the same color indicate proteins enriched in the same KEGG pathway.

A homology-based predicted protein association network was constructed for MSTN ligands and their putative receptors in *T. ovatus* using STRING based on zebrafish homologs (Figure 8). All identified MSTN ligands and putative receptor proteins were represented in the network and showed predicted associations with other network components. Several TGF-β superfamily-related proteins, including gdf11 and gdf10a, were also present in the network. Overall, the predicted associations suggest that MSTN-related proteins may be linked to a broader TGF-β signaling network. However, because these associations were inferred from zebrafish homologs, they should be regarded as candidate relationships requiring experimental validation in *T. ovatus*.

## 3. Discussion

The MSTN signaling system, as an important ligand–receptor branch of the TGF-β superfamily, plays important regulatory roles in skeletal muscle growth, tissue development, metabolism, and physiological homeostasis across vertebrates. MSTN has been widely recognized as a negative regulator of skeletal muscle mass, and its functional importance has been demonstrated in both mammals and teleosts [1]. In this study, we systematically identified and characterized MSTN-related genes in *T. ovatus*, revealing nine members, including two MSTN ligand genes (*ToMSTNa* and *ToMSTNb*) and seven putative receptor genes (*ToACVR1A*, *ToACVR1Ba*, *ToACVR1Bb*, *ToACVR2Aa*, *ToACVR2Ab*, *ToACVR2Ba*, and *ToACVR2Bb*). The presence of two MSTN paralogs is consistent with the duplicated MSTN pattern reported in teleost fishes [7,16]. Together, these findings provide valuable genetic resources for understanding the evolution of MSTN-related genes in *T. ovatus*.

Conserved motif and domain analyses, together with gene structure and synteny results, provided further insight into the evolution of MSTN-related genes in *T. ovatus*. In the present study, *ToMSTNa* and *ToMSTNb* displayed highly similar motif compositions and retained the characteristic TGF-β superfamily-associated architecture, including regions required for precursor processing and mature ligand formation [17,18], indicating that these two ligands retain conserved structural features of MSTN proteins. Previous studies have shown that MSTN is synthesized as a latent precursor in which the N-terminal prodomain remains associated with the C-terminal mature ligand, thereby maintaining the growth factor in an inactive state prior to proteolytic activation [17,19]. By contrast, the putative receptor proteins contained typical activin receptor-related domains. Members of the ACVR1 subgroup possessed the characteristic GS region together with intracellular serine/threonine kinase domains, whereas ACVR2 subgroup members contained conserved receptor kinase-related domains, supporting their potential involvement in ligand recognition and downstream signal transduction [20,21,22]. Gene structure analysis further showed that MSTN ligand genes and their putative receptor genes in *T. ovatus* exhibited distinct exon–intron organizations. *ToMSTNa* and *ToMSTNb* displayed relatively compact structures, each containing three exons, whereas the receptor genes showed more complex and variable structures with longer genomic spans. Such differences suggest that MSTN ligands and their receptors may have undergone different evolutionary constraints during gene family diversification [22,23].

In addition, the nine MSTN-related genes were unevenly distributed across seven chromosomes of *T. ovatus*, with LG7 and LG12 each containing two MSTN-related genes, whereas LG3, LG8, LG9, LG19, and LG22 each harbored only one. This chromosomal distribution showed no clear association with sequence similarity or phylogenetic classification, suggesting that most MSTN-related genes are unlikely to have originated from recent tandem duplication events. Instead, their distribution pattern may reflect ancient duplication and genome rearrangement during teleost genome evolution [24,25]. Because young duplicate genes often arise in adjacent regions on the same chromosome [26], the absence of extensive tandem clustering among MSTN-related genes suggests that most members may have originated from ancient duplication events rather than recent tandem duplication. Interspecific synteny analysis further showed that six of the eight analyzed MSTN-related genes had conserved syntenic relationships with zebrafish homologs, suggesting that the MSTN-related gene family in *T. ovatus* is evolutionarily conserved [27].

The homology-based predicted protein association network suggested potential associations between MSTN-related proteins and components of the broader TGF-β superfamily signaling pathway. These predicted associations are broadly consistent with the canonical MSTN signaling model, in which MSTN/GDF8 binds to activin type II receptors, particularly ACVR2A or ACVR2B, and subsequently recruits type I receptors such as ACVR1B/ALK4 to form active receptor complexes [4,20]. Activation of these receptor complexes can trigger downstream SMAD2/3 signaling, thereby regulating muscle growth and related physiological processes [28]. However, because the network was inferred from zebrafish homologs, these predicted associations should be considered candidate relationships that require experimental validation in *T. ovatus*.

Previous studies have shown that teleost mstn genes often display broader and more diverse tissue expression patterns than their mammalian counterparts, suggesting functional divergence after gene duplication [29,30,31]. In the present study, MSTN ligands and their putative receptor genes were expressed in multiple tissues of *T. ovatus*, but their expression patterns differed among members. *ToMSTNa* was highly expressed in muscle, which is consistent with previous reports suggesting a conserved association between MSTN expression and skeletal muscle growth regulation in fish [32,33]. By contrast, *ToMSTNb* showed relatively high expression in brain, resembling the tissue divergence of duplicated mstn paralogs reported in zebrafish and Nile tilapia, in which one paralog showed preferential expression in non-muscular tissues such as the brain [8,10,30]. Compared with the two MSTN ligands, the putative receptor genes displayed more diverse tissue specificity, with *ToACVR1A*, *ToACVR1Ba*, *ToACVR2Aa*, and *ToACVR2Ab* showing relatively high expression in blood, *ToACVR2Ba* in brain and fin, and *ToACVR1Bb* and *ToACVR2Bb* in fin and ovary. The relatively high expression of several receptor genes in blood may indicate that activin/TGF-β-related signaling is involved in systemic physiological regulation rather than being restricted to skeletal muscle regulation. These findings suggest that the MSTN ligand–receptor system in *T. ovatus* may be involved in tissue-specific physiological processes beyond skeletal muscle.

This study further analyzed the expression patterns of MSTN-related genes in the brain, intestine, and liver of large and small *T. ovatus*. *ToMSTNa* showed a trend toward higher expression in the brain and intestine of small individuals than in large individuals. Previous studies have shown that teleost MSTN genes can exhibit tissue-specific expression patterns beyond skeletal muscle, and duplicated MSTN paralogs may undergo functional divergence among different tissues [31,33]. Because MSTN is generally considered a negative regulator of somatic growth, the observed expression trend of *ToMSTNa* may suggest a potential association between MSTN-related signaling and growth variation [1,34]. However, its higher expression in brain and intestine, rather than only in skeletal muscle, suggests that *ToMSTNa* may be associated with growth variation through tissue-specific expression patterns involving non-muscular tissues [8,10]. In addition, *ToACVR1A* and *ToACVR2Bb* showed trends toward higher expression in the liver of small individuals than in large individuals. Activin receptor signaling has been reported to regulate body mass growth and muscle development in teleost fish [35,36], suggesting a potential relationship between the expression patterns of these receptors and growth-related processes. Considering the central role of the liver in nutrient metabolism and energy homeostasis [37], these expression trends may also indicate a potential relationship between MSTN-related signaling and metabolic processes during growth. Together, these results suggest a potential association between the tissue-specific expression patterns of MSTN-related genes and growth variation in *T. ovatus*. Further functional validation, such as RNA interference, overexpression, receptor inhibition, or SMAD2/3 phosphorylation analysis, will be required to clarify whether these expression differences directly contribute to growth regulation.

## 4. Materials and Methods

### 4.1. Ethical Statement

The Committee of the South China Sea Fisheries Research Institute, Chinese Academy of Fisheries Sciences (no. SCSFRI96-263), approved the animal protocols, and all experiments were performed under the applicable standards.

### 4.2. Identification of MSTN-Related Genes in T. ovatus

**Figure 9:**
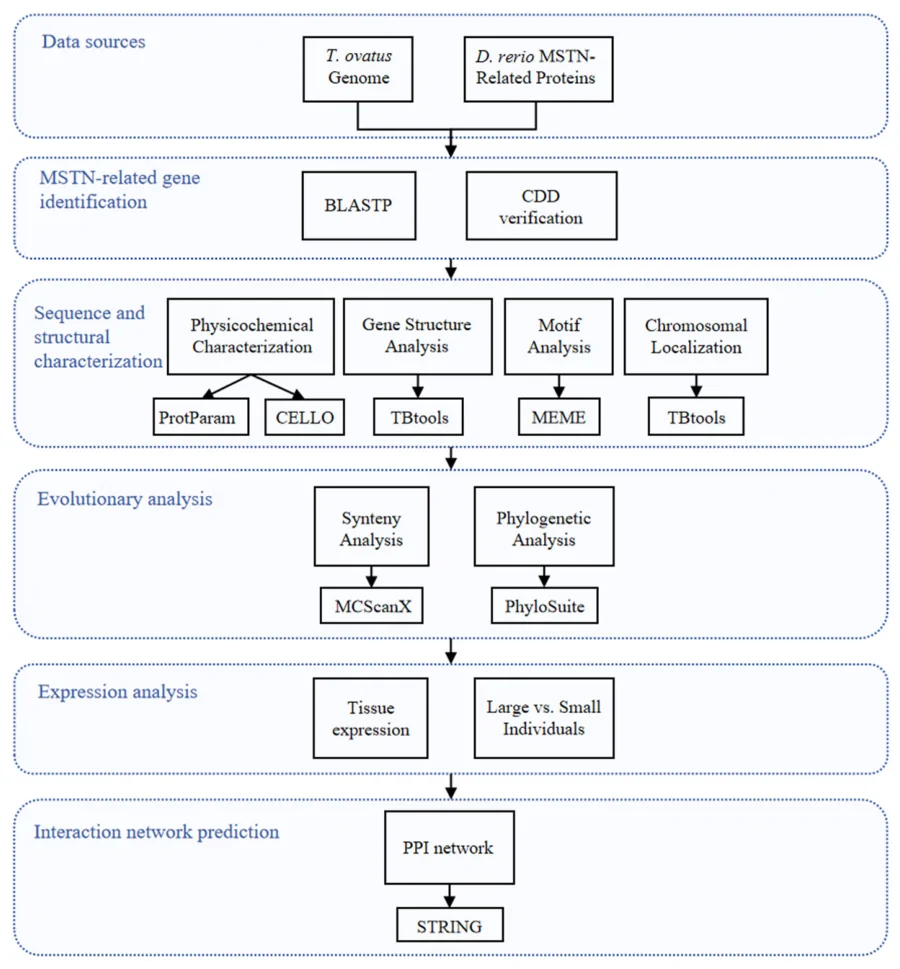
Schematic workflow for the identification and characterization of MSTN-related genes in *T. ovatus*.

The overall workflow for the identification and characterization of MSTN-related genes in *T. ovatus* is shown in Figure 9. Genomic data for *T. ovatus* were obtained from the NCBI GenBank database (genome assembly accession: GCA_900231065.1). The MSTN and putative receptor protein sequences of zebrafish (*Danio rerio*) were retrieved from the National Center for Biotechnology Information (NCBI) database (https://www.ncbi.nlm.nih.gov/assembly/, accessed on 15 April 2026). To identify MSTN genes and their putative receptor genes in *T. ovatus*, the corresponding amino acid sequences of D. rerio were used as queries to perform local BLASTP searches against the *T. ovatus* genome database, with an E-value threshold of 2 × 10^−5^. Candidate sequences were further examined using the NCBI Batch Web CD-Search Tool (https://www.ncbi.nlm.nih.gov/Structure/bwrpsb/bwrpsb.cgi, accessed on 16 April 2026) to confirm the presence of conserved MSTN- or receptor-related domains (an E-value threshold of <0.01 was applied in CDD searches). The nucleotide and deduced amino acid sequences of the identified genes were then manually checked to remove redundant sequences and verify the integrity of the coding sequences and conserved domains before subsequent analyses. The physicochemical properties of the encoded proteins, including amino acid length, molecular weight, theoretical isoelectric point and grand average of hydropathicity, were predicted using ProtParam (https://web.expasy.org/protparam/, accessed on 17 April 2026). Subcellular localization was predicted using CELLO v.2.5 (https://combio.life.nctu.edu.tw/cello/, accessed on 20 April 2026).

### 4.3. Analysis of Conserved Motifs, Structural Domains, and Gene Structures

Protein sequences of *T. ovatus* MSTN ligands and their putative receptors were submitted to MEME (https://meme-suite.org/meme/, accessed on 25 April 2026) for conserved motif analysis. The analysis was performed in classic mode, allowing any number of motif repetitions, with the maximum number of motifs set to 20. Gene structures of *T. ovatus* MSTN genes and their putative receptor genes were analyzed based on genome annotation files, including the organization of coding sequences (CDSs) and untranslated regions (UTRs). Conserved domain information, motif composition, and gene structure data were integrated and visualized using TBtools-II v2.507 [38].

### 4.4. Chromosomal Localization and Collinearity Analysis

Based on the gene annotation information in the GFF file of *T. ovatus*, the chromosomal positions of MSTN-related genes and the corresponding chromosome lengths were extracted using TBtools. The physical distribution of MSTN genes on chromosomes was then visualized using the MapInspect function in TBtools. For collinearity analysis, the GFF files of *T. ovatus* and zebrafish were imported into TBtools. Gene duplication events and syntenic regions were identified using MCScanX v1.0.0 [39], and the results were visualized with TBtools.

### 4.5. Phylogenetic Analysis

Phylogenetic analyses were performed to support the annotation and classification of the identified MSTN proteins and their putative receptors in *T. ovatus*. Amino acid sequences of MSTN ligands and their putative receptors from other vertebrates were downloaded from the NCBI database and used to construct phylogenetic trees. The sequences used in this analysis are listed in Table S1. Multiple sequence alignment was performed using MAFFT v7.505 implemented in PhyloSuite v1.2.3. A maximum likelihood (ML) phylogenetic tree was constructed using IQ-TREE v2.2.0, with the best-fit substitution model selected automatically. The reliability of each node was assessed using 1000 bootstrap replicates. Other parameters were kept at their default settings. The MSTN-related genes in *T. ovatus* were named according to their phylogenetic clustering patterns with known homologs and the presence of conserved functional domains. The phylogenetic tree was visualized using Evolview v3.

### 4.6. Sample Collection and Transcriptome Expression Analysis

#### 4.6.1. Sample Collection for Growth-Difference Analysis

For growth-difference analysis, *T. ovatus* were collected from the Tropical Aquaculture Research and Development Center of the South China Sea Fisheries Research Institute, Chinese Academy of Fishery Sciences. All fish originated from the same batch of fry produced by induced spawning in May 2021 and were reared in cage culture at Xincun fish rafts under the same feeding regime and environmental conditions. On 1 December 2023, approximately 2000 fish from this cohort were manually screened according to growth performance, and 12 fast-growing and 12 slow-growing individuals were selected. The fast-growing and slow-growing groups were designated as L and S, respectively, and their body weights were approximately 2 kg and 1 kg, respectively. For the individuals used for RNA sequencing (RNA-seq) analysis, individual body weight, body length, and condition factor were recorded and are provided in Table S4. Six individuals from each group were used for RNA-seq and subsequent expression analysis (*n* = 6 per group), while the remaining individuals were used for other analyses. After selection, the fish were transferred to a new cage and acclimated for 7 days before sampling. Whole brain, liver, and intestine tissues were collected from each fish, rinsed with physiological saline to remove blood and impurities, blotted dry with absorbent paper, and divided into two portions, with each portion placed into a separate 2 mL cryogenic tube. Therefore, two brain samples, two liver samples, and two intestine samples were obtained from each fish. All samples were immediately frozen in liquid nitrogen and stored for subsequent analysis.

#### 4.6.2. RNA Extraction and Sequencing Analysis

Total RNA was extracted from the collected tissue samples using TRIzol reagent (Invitrogen, Thermo Fisher Scientific, Waltham, MA, USA) according to the manufacturer’s instructions. RNA integrity was assessed by 1% agarose gel electrophoresis, and RNA concentration and purity were determined using a NanoDrop 2000 spectrophotometer (Thermo Fisher Scientific, USA). Qualified RNA samples were then used for library construction and sequenced on the Illumina NovaSeq 6000 platform (Illumina, San Diego, CA, USA). Raw reads were filtered using fastp (v0.23.4) to obtain high-quality clean reads for subsequent analysis.

#### 4.6.3. Expression Analysis of MSTN-Related Genes

Two independent transcriptome datasets were used to analyze the expression patterns of MSTN-related genes. For the basal tissue-expression analysis, transcriptomic data from 12 tissues of adult *T. ovatus*, including intestine, liver, muscle, brain, spleen, fin, gill, kidney, stomach, blood, testis, and ovary, were used, with three biological replicates for each tissue (*n* = 3). For the growth-associated expression analysis, an independent transcriptome dataset comprising brain, intestine, and liver samples from large (L) and small (S) individuals was used, with six biological replicates per group (*n* = 6). For the large-versus-small comparison, the S group was used as the reference group for fold-change calculation. The RNA-seq data used for the large-versus-small comparison have been deposited in the NCBI Sequence Read Archive (SRA) under SRA Study accession SRP722126. Raw sequencing reads were filtered using fastp (v0.23.4) to obtain high-quality clean reads. The clean reads were aligned to the *T. ovatus* reference genome using HISAT2 (v2.0.5). The mapped reads were assembled using StringTie (v1.3.3b), and gene-level read counts were generated using featureCounts (v1.5.0-p3). Gene expression levels were quantified as FPKM for expression profiling. The RNA-seq datasets were used to characterize the tissue-specific and growth-associated expression patterns of previously identified MSTN-related genes. For expression profiling and hierarchical clustering, FPKM values from individual biological replicates were log_2_-transformed and normalized using gene-wise Z-score transformation. Heatmaps were generated to visualize the expression patterns of MSTN-related genes across different tissues and growth groups.

### 4.7. PPI Network Analysis of MSTN-Related Genes

A homology-based predicted protein association network of *T. ovatus* MSTN ligands and their putative receptors was constructed using the STRING database based on homologous protein sequences from zebrafish. The maximum number of interactors was set to 50 for the first shell and 20 for the second shell, and the minimum required interaction score was set to 0.400.

## 5. Conclusions

This study systematically identified and characterized nine MSTN-related genes in *T. ovatus*, including two MSTN ligands and seven putative receptor genes. Integrated analyses of gene structure, conserved motifs, chromosomal localization, phylogenetic and syntenic relationships, and tissue expression patterns indicated evolutionary conservation and expression divergence among these genes. Tissue expression analysis showed that *ToMSTNa* was relatively highly expressed in muscle, whereas *ToMSTNb* was relatively highly expressed in brain. Comparative expression analysis between large and small individuals showed distinct expression patterns of several MSTN-related genes, including *ToMSTNa*, *ToACVR1A*, and *ToACVR2Bb*. These findings suggest a potential association between MSTN-related gene expression and growth variation in *T. ovatus*. Further experimental validation is required to determine the functional roles of these genes and their potential involvement in growth regulation.

## Figures and Tables

**Table 1:** Identified MSTN-related genes and physicochemical properties of their encoded proteins in *T. ovatus*.

Gene	Chr	Size (aa)	Molecular Weight	PI	Instability Index	Aliphatic Index	Grand Average of Hydropathicity	Subcellular Localization
*ToMSTNa*	LG7	370	40,895.25	6.1	64.77	75.65	−0.427	Nuclear
*ToMSTNb*	LG19	369	41,806.96	6.78	57.38	83.79	−0.393	Nuclear
*ToACVR1A*	LG12	488	54,470.64	7.87	43.48	87.13	−0.2	Mitochondrial
*ToACVR1Ba*	LG3	364	41,682.87	7.67	43.07	90.03	−0.299	Mitochondrial
*ToACVR1Bb*	LG8	483	54,330.26	6.42	44.58	91.47	−0.175	Plasma Membrane
*ToACVR2Aa*	LG9	528	59,593.1	5.75	50.86	82.73	−0.259	Cytoplasmic
*ToACVR2Ab*	LG7	529	59,180.98	5.89	57.09	85.12	−0.161	Cytoplasmic
*ToACVR2Ba*	LG12	504	56,675.95	5.72	48.07	79.88	−0.277	Cytoplasmic
*ToACVR2Bb*	LG22	505	57,348.1	9.06	47.85	72.22	−0.602	Extracellular

## Data Availability

The original contributions presented in this study are included in the article/Supplementary Material. Further inquiries can be directed to the corresponding author.

## Supplementary Materials

The following supporting information can be downloaded at: https://www.mdpi.com/article/10.3390/ijms27188061/s1.
