# Supplementary material for: Genome-Wide Characterization of the Myostatin Gene (MSTN) Ligand–Receptor System and Its Expression Patterns Associated with Growth Variation in Trachinotus ovatus
Source: Int J Mol Sci. 2026 Sep 10;27(18):8061. doi: 10.3390/ijms27188061 (PMC13606707; doi:10.3390/ijms27188061)
Supplement: Supplementary file 1 [file ijms-27-08061-s001.zip › Figure S1.pdf]

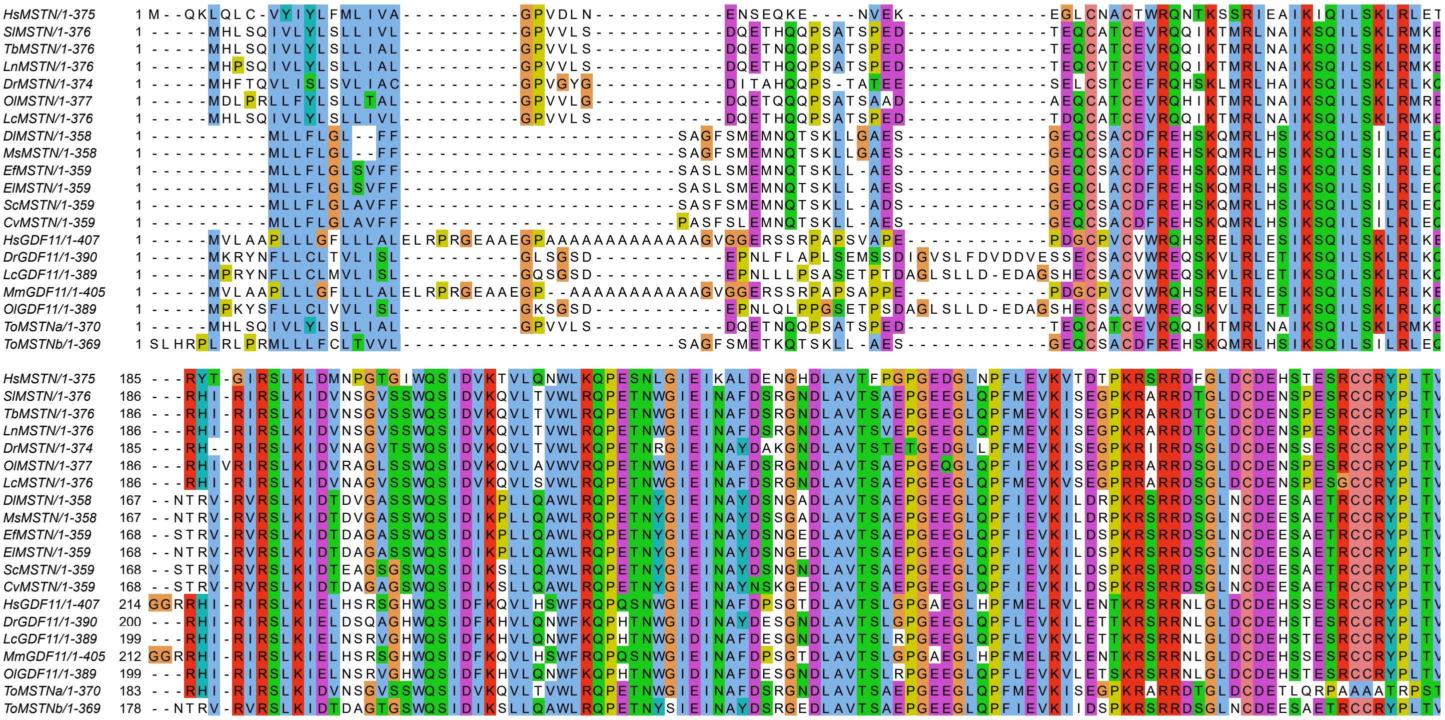

Figure S1. Multiple sequence alignment of MSTN ligand proteins from *T. ovatus* and other representative species.
