# Supplementary material for: Genome-Wide Characterization of the Myostatin Gene (MSTN) Ligand–Receptor System and Its Expression Patterns Associated with Growth Variation in Trachinotus ovatus
Source: Int J Mol Sci. 2026 Sep 10;27(18):8061. doi: 10.3390/ijms27188061 (PMC13606707; doi:10.3390/ijms27188061)
Supplement: Supplementary file 1 [file ijms-27-08061-s001.zip › Figure S2.pdf]

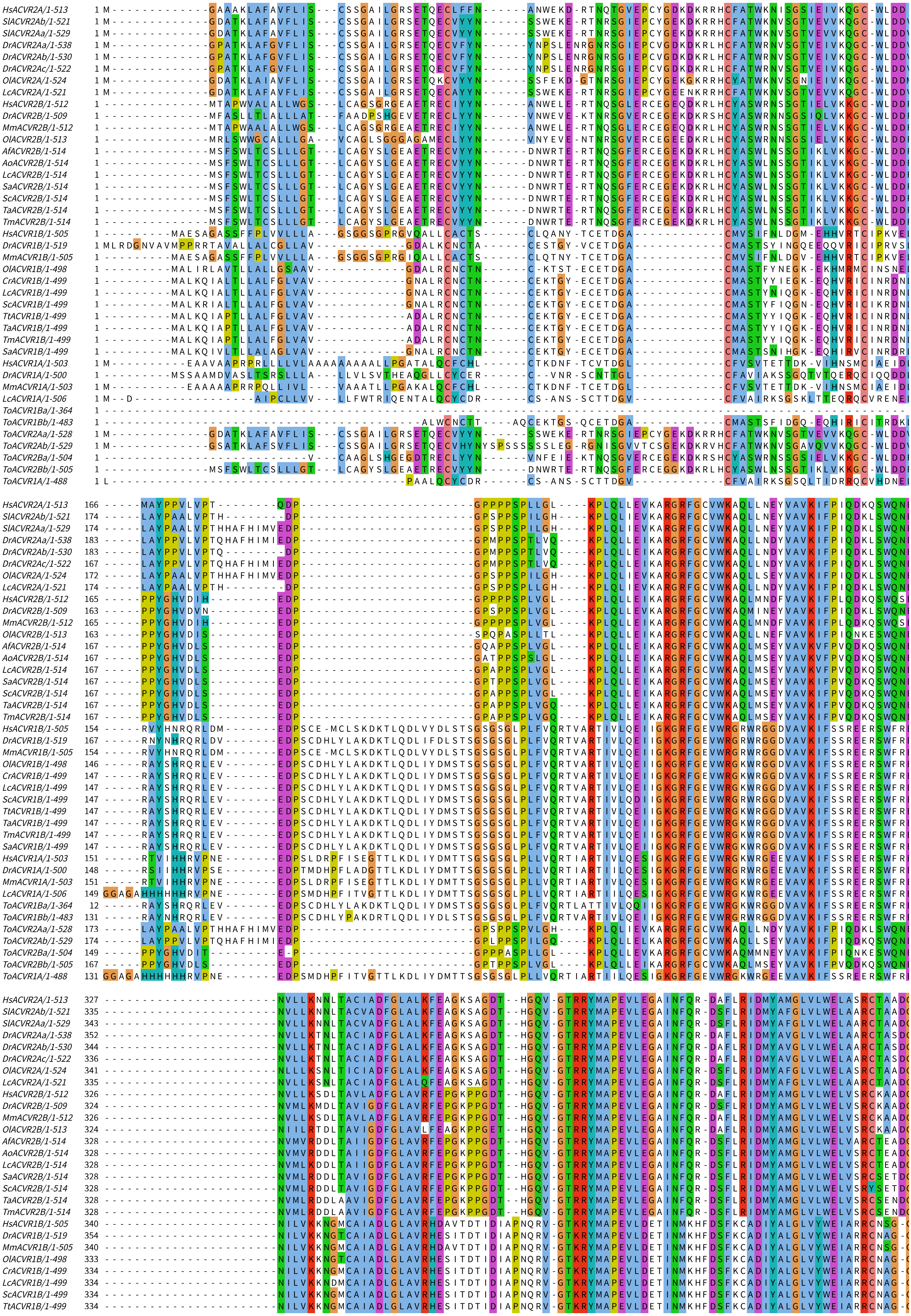
HsACVR2A/1-513
SlACVR2Ab/1-521
SlACVR2Aa/1-529
DrACVR2Aa/1-538
DrACVR2Ab/1-530
DrACVR2Ac/1-522
OlACVR2A/1-524
LcACVR2A/1-521
HsACVR2B/1-512
DrACVR2B/1-509
MmACVR2B/1-512
OlACVR2B/1-513
AfACVR2B/1-514
AoACVR2B/1-514
LcACVR2B/1-514
SaACVR2B/1-514
ScACVR2B/1-514
TaACVR2B/1-514
TmACVR2B/1-514
HsACVR1B/1-505
DrACVR1B/1-519
MmACVR1B/1-505
OlACVR1B/1-498
CrACVR1B/1-499
LcACVR1B/1-499
ScACVR1B/1-499
TtACVR1B/1-499
TaACVR1B/1-499
TmACVR1B/1-499
SaACVR1B/1-499
HsACVR1A/1-503
DrACVR1A/1-500
MmACVR1A/1-503
LcACVR1A/1-506
ToACVR1Ba/1-364
ToACVR1Bb/1-483
ToACVR2Aa/1-528
ToACVR2Ab/1-529
ToACVR2Ba/1-504
ToACVR2Bb/1-505
ToACVR1A/1-488

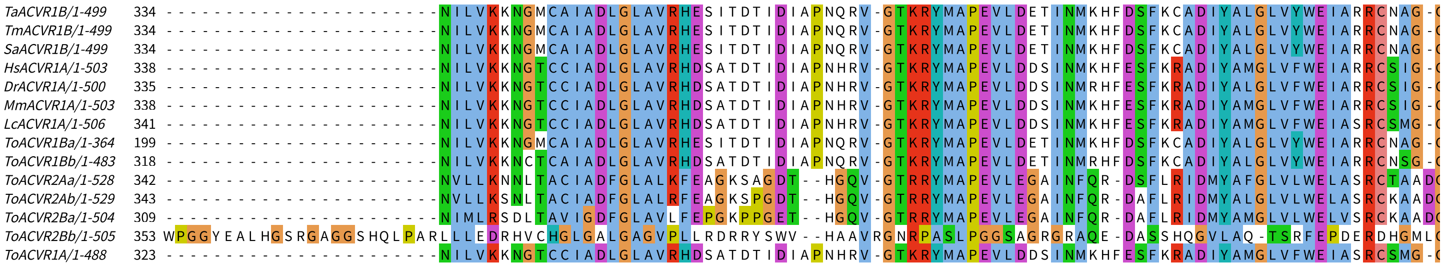

Figure S2. Multiple sequence alignment of ACVR proteins from *T. ovatus* and other representative species.
